# Perceptions of hospital feeding practices among mothers of infants with severe pneumonia in Malawi: a qualitative descriptive study

**DOI:** 10.1136/bmjopen-2024-094793

**Published:** 2025-06-08

**Authors:** Nadia E Hoekstra, Dalton M Craven, Mercy Tsidya, Annie Thom, Agatha Bula, Marieke van der Zalm, Tisungane Mvalo, Eric D McCollum

**Affiliations:** 1 UNC Project-Malawi, Lilongwe, Central Region, Malawi; 2 Global Program in Pediatric Respiratory Sciences, The Johns Hopkins University School of Medicine, Baltimore, Maryland, USA; 3 Pediatrics, The University of North Carolina at Chapel Hill School of Medicine, Chapel Hill, North Carolina, USA; 4 Department of Paediatrics and Child Health, Faculty of Medicine and Health Sciences, Stellenbosch University, Desmond Tutu TB Centre, Cape Town, South Africa; 5 International Health, Johns Hopkins Bloomberg School of Public Health, Baltimore, Maryland, USA

**Keywords:** Child, Tropical medicine, Paediatric thoracic medicine, QUALITATIVE RESEARCH, Respiratory infections, Nutrition

## Abstract

**Abstract:**

**Objective:**

To determine caregiver knowledge of, attitudes towards, and perceptions of feeding practices for infants admitted to a tertiary referral hospital with severe pneumonia, and to identify community feelings about nasogastric tube feeding in Malawi.

**Setting:**

The paediatric ward of a government tertiary referral hospital in Lilongwe, Malawi.

**Methods:**

From March through April 2023, we conducted in-depth interviews with 14 mothers of infants 0–12 months of age hospitalised with severe pneumonia who had been enrolled in an observational study evaluating feeding and swallowing in breastfed infants. In-depth interviews assessed mothers’ attitudes towards hospital feeding practices including nasogastric tube feeding, along with community perceptions of nasogastric tubes. Data were analysed using a thematic analysis approach to assess themes and subthemes of transcripts.

**Results:**

Mothers understood that children with pneumonia are at risk of ‘choking’ during oral feeding; however, they had substantial worries about both withholding breastfeeding and providing nasogastric feeds to their infants through a nasogastric tube. Community perceptions of nasogastric tubes were widely negative and included beliefs that nasogastric tubes cause children to ‘choke’ and die and that medical providers want to harm children. Mothers held strong beliefs about the benefits of breastfeeding.

**Conclusion:**

There are alarming misconceptions in the community around nasogastric tubes and the intentions of medical providers. This leads to maternal concerns about this hospital feeding practice, poor adherence to medical recommendations, and mistrust in the broader healthcare system. To improve hospital outcomes of children with severe pneumonia, caregiver and community education is needed.

**STRENGTHS AND LIMITATIONS OF THIS STUDY**

- In-depth interviews included mothers of infants who were admitted to a government tertiary referral hospital and enrolled in an observational study evaluating feeding and swallowing in breastfed infants with severe pneumonia.
- Mothers of infants who required different levels of respiratory support and feeding routes (ie, oral or nasogastric) during hospitalisation were interviewed.
- Interviews were conducted by trained Malawian facilitators in the local language of Chichewa and data were collected until thematic saturation was reached.
- This study is limited in that the findings may not represent the beliefs of all mothers in Malawi, and medical providers were not interviewed about their beliefs, which may have influenced the education they provided to mothers and thus affected mothers’ responses.

## Introduction

 Pneumonia remains the single largest infectious cause of death in children globally.^1^ The burden of childhood pneumonia is high in low- and middle-income countries (LMICs), particularly those in South Asia and sub-Saharan Africa where resources are limited. In 2021, approximately 502 000 deaths from pneumonia occurred in sub-Saharan Africa.^2^ In Malawi, one of the poorest countries in this region, the mortality rate for infants hospitalised with pneumonia is approximately 4%.^3^

Some pneumonia-related deaths in LMICs may be attributed to aspiration during oral feeding.^4^ Children experiencing respiratory distress from pneumonia have an increased risk of aspiration from swallow dysfunction.^5^ Emerging treatments for pneumonia in low-resource settings, such as non-invasive ventilation with continuous positive airway pressure (CPAP),^6^ may further contribute to swallowing difficulties and aspiration risk during feeding.^7 8^ To reduce the risk of life-threatening aspiration events in children hospitalised with severe respiratory distress, particularly those requiring non-invasive ventilation, medical providers may temporarily recommend nasogastric tube feeding rather than oral feeding.^9^ Nasogastric tubes are narrow, plastic tubes inserted through the nose, down the throat and oesophagus, into the stomach, and used to deliver liquid nutrition and medications directly into the stomach.

Current World Health Organisation (WHO) guidelines on child nutrition recommend exclusive, on-demand breastfeeding for infants during the first 6 months of life and continued breastfeeding up to 2 years.^10^ For emerging pneumonia treatments to be safe, feasible and effective, ensuring caregiver acceptance and adherence to temporary alternative hospital feeding practices is essential. In a trial conducted in Malawi evaluating the use of bubble CPAP non-invasive ventilation for treatment of severe pneumonia in children, caregivers may have orally fed their children despite medical providers having inserted nasogastric tubes for feeding and medications.^4^ Mothers in sub-Saharan Africa who exclusively breastfeed their children indicate that family and community members influence their choice of feeding method at home.^11^ Caregiver and community member perceptions of nasogastric tube feeding in sick, hospitalised children are largely unknown in Africa. We aimed to determine caregiver knowledge of, attitudes towards, and perceptions of feeding practices for infants admitted to a government tertiary referral hospital with severe pneumonia, and to identify community feelings about nasogastric tube feeding in Malawi.

## Methods

### Setting

Kamuzu Central Hospital (KCH), located in Lilongwe, Malawi, is a tertiary referral hospital serving the Central Region of the country. On average, approximately 40 infants aged 0–5 months are admitted to KCH each month with severe pneumonia.^3^

At KCH, infants and children who have severe respiratory distress and require bubble CPAP are not permitted to feed orally. As a result, breastfeeding mothers are instructed by medical providers to cease direct breastfeeding. Instead, a nasogastric tube is inserted to facilitate feeding. Mothers are advised to express breast milk and administer it through the nasogastric tube, as formula is not available in this setting.

### Study design

This descriptive qualitative study was conducted at KCH from 28 March through 19 April 2023. We conducted in-depth interviews with 14 mothers of breastfed infants 0–12 months of age who were admitted to the paediatric ward with WHO-defined severe pneumonia^12^ and enrolled in an observational study that evaluated feeding and swallowing in breastfed infants with severe pneumonia (unpublished).

Prior to enrolment in the parent observational study, mothers of eligible infants were informed about the study and those who showed interest provided written informed consent.

Mothers of infants admitted to the paediatric ward at KCH who were participating in the parent observational study were chosen for interviews through opportunistic and convenience sampling. Specifically, mothers who were present at their infant’s bedside during the study period were eligible to participate. We approached mothers of infants who required bubble CPAP, as well as those whose infants received either conventional low flow oxygen or no respiratory support. Infant age, sex and bubble CPAP use during hospitalisation were documented, alongside maternal demographic data, including age, number of children and highest education level achieved.

Interviews were held in a private room at KCH and conducted in the local language of Chichewa by a facilitator trained in qualitative research (MT, AT). The interview guide was developed by the research team, reviewed by local experts and piloted prior to interviews (online supplemental files 12). Mothers were asked open-ended questions about their experiences during their child’s treatment and the information they learned from hospital staff. They were asked whether they were told by a medical provider to stop breastfeeding their child and were invited to provide their opinions on feeding practices in the hospital, including breastfeeding and nasogastric tube feeding. Mothers were also asked to discuss beliefs they had heard from community members about feeding children using nasogastric tubes.

Each interview lasted 10–30 min and was audio-recorded by the facilitator. Recordings of interviews were transcribed and translated from Chichewa to English.

### Data management and analytics

Data were analysed following a thematic analysis approach^13^ with NVivo V.14 software. An initial codebook was developed from the interview guide using pre-defined themes. Following each interview, members of the research team read the transcript in full and met to discuss emerging themes. Codes were added to the codebook iteratively, and the final version was established through consensus by the research team. Each transcript was then coded by two of three independent reviewers (NEH, DMC and MT). The team met weekly to review and reconcile all areas of discrepancy until agreement of the coded text was reached via consensus.

The team identified themes and subthemes, and then used Microsoft Excel to construct matrices summarising them. All themes were saturated after 14 interviews. Findings were then synthesised using qualitative memo writing. Descriptive statistics were used to analyse quantitative variables.

### Patient and public involvement

Due to resource limitations, it was not feasible to involve patients in the study design, conduct, reporting or dissemination. Members of the public including the KCH Department of Paediatrics, the KCH Research Committee and the Lilongwe District Health Office Research Committee were involved in the design, conduct, reporting and dissemination of this research.

## Results

### Demographics

A total of 14 mothers participated in interviews, while two declined. Table 1 reports the demographic characteristics of the participating mothers and their infants with severe pneumonia.

**Table 1:** Demographics of mothers and their infants with severe pneumonia

Mothers (n=14)	
Age in years, median (IQR)	29.5 (25.0–32.7)
Number of children, median (IQR)	2 (1–3)
Enrolled in secondary school or higher education, n (%)	8 (57.1)
Infants (n=14)	
Age in months, median (IQR)	5.0 (2.0–8.7)
Female sex, n (%)	7 (50.0)
Required bubble CPAP, n (%)	7 (50.0)

CPAP, continuous positive airway pressure.

Five mothers (35.7%) reported being advised by a medical provider to discontinue direct breastfeeding. In these cases, providers recommended the insertion of a nasogastric tube and feeding the infant with expressed breast milk via the tube.

### Major themes

Several major themes emerged from in-depth interviews with participants. These included: substantial maternal knowledge about feeding problems in children with pneumonia; moderate understanding of the importance of temporarily withholding breastfeeding in some children with pneumonia; substantial worry about both withholding breastfeeding and administering nasogastric feeds through a nasogastric tube; and consistent beliefs about the benefits of breastfeeding, even for children who may be too ill to feed safely by mouth.

#### Knowledge about oral feeding problems in children with pneumonia

Most participants expressed understanding that children who have breathing difficulties can have problems with breastfeeding by mouth. Some participants recognised that their own children were having trouble breastfeeding, while others had heard from others that children with pneumonia can experience oral feeding problems.

Yesterday, all my breasts were swollen of milk because he was not breastfeeding. They are currently still swollen because he is not breastfeeding the way he normally does. (Participant 2)

Those that heard about feeding problems in children with pneumonia cited various sources including medical providers, community members and other mothers. One participant shared what she had heard when she initially presented to another hospital to seek care for her child:

I heard that when a child has pneumonia, he has difficulty breathing and even feeding itself is a challenge. (Participant 12)

Another participant shared that community members told her that children with pneumonia experience a sensation in their ribs when they are breastfeeding:

Yes, they [infant with pneumonia] have trouble feeding because when they are sucking the breast, they feel prickly in the ribs, that is what they [community members] said. (Participant 2)

#### Understanding of reasons to withhold oral feeding

Each participant was asked why some children with pneumonia are not allowed to breastfeed while hospitalised. A few participants who had not been advised to stop breastfeeding were unaware of the reasons why some mothers are instructed to do so. However, most participants correctly identified that providers advise some mothers to stop breastfeeding their children with breathing difficulties because they may ‘choke’, a term that implies airway obstruction or aspiration during feeding.

One participant specifically mentioned that milk may flow into the wrong place if the child continues to breastfeed with breathing difficulty:

Maybe because as they are breastfeeding, they might have trouble breathing with the milk and the milk might end up going in another passage other than the throat. (Participant 8)

Another participant highlighted that children with pneumonia are treated with oxygen and that sometimes doctors have alternative ways to feed children on oxygen by using tubes:

Maybe because the child cannot manage to suck the breast on their own. That is where some are told to express into a cup and then give the child. At times, you wait for the child to be given some oxygen so that they feel a little better before you can feed them. At times, the doctors here at the hospital have their own ways of feeding the child through tubes. (Participant 2)

One participant explained that children may improve faster if they are not breastfeeding:

My understanding was that the provider wanted my child to be healed faster because if I had continued breastfeeding, we would have stayed long in the hospital. (Participant 11)

Another participant reported the belief that children who are not taking in enough breast milk due to breathing difficulties may get worse due to not receiving enough food:

The other one could be that the intake of food by the child may not be enough because he can stop while hungry but because he has difficulty consuming, he can just stop and that can make the illness worse because he is not eating. (Participant 12)

#### Worries about withholding breastfeeding

Regardless of whether they were told to stop breastfeeding during the hospitalisation, we asked participants about their feelings about withholding breastfeeding if instructed by a medical provider. Several participants expressed worries about stopping breastfeeding because of the impact it may have on the child’s health.

One participant who was asked to stop breastfeeding during the hospitalisation shared her worries at the time:

The child’s health depends on breastfeeding and I do not know what can happen to her if she stops breastfeeding. (Participant 3)

A participant who was not told to stop breastfeeding discussed that small children depend on breast milk and thus should not stop breastfeeding. She mentioned that if there is an alternative way to breastfeed the child, this would be preferred:

The child should not stop breastfeeding because a small child relies on breastfeeding most times. When the child starts taking solid food is when they can be given other food stuffs. But, for small children, they depend on the breast and so they should not stop breastfeeding. If there is an alternative way of breastfeeding the child, that would be better, but the child should not completely stop breastfeeding. (Participant 2)

#### Concerns about nasogastric tube feeding

The overwhelming majority of participants had concerns about the use of nasogastric tubes and several reported negative perceptions in the community. Multiple participants stated that they thought nasogastric tubes can cause children to ‘choke’:

I feel that the child can be at risk of choking because as I said, the food is supposed to go through the mouth but for food to go through the tube, it is not healthy for the child. (Participant 7)

Even participants who were not told to feed their child with a nasogastric tube during the hospitalisation had negative opinions. When asked about how she would feel if the providers had recommended nasogastric tube feeding, one participant expressed concerns about provider malintent:

I can be concerned because I may feel that the health providers want to harm my child. (Participant 1)

Only one participant had a positive perception of nasogastric tube feeding after she experienced her child being fed this way.

I felt good because I knew that the doctors want my child to be assisted. I was not worried because it was better when he was put on nasogastric tube compared to the time when he was refusing to breastfeed and crying persistently. (Participant 12)

Before receiving an explanation from a medical provider, this participant initially assumed that her child would be given formula through a nasogastric tube instead of being breastfed.

My only concern was that when my child will resume breastfeeding, the breast milk will be sour or not user friendly because I would stay for long without him breastfeeding. I also thought that my breasts will swell because I thought we were to use formula but then they said we were to express the breast milk and pour in the nasogastric tube so I was okay with that. (Participant 12)

Most participants shared that their community members also have negative opinions about nasogastric tubes. Several participants stated that they heard from others that children die from nasogastric tubes:

They say that the tube can kill the child. (Participant 9)

When questioned on how nasogastric tubes can kill children, this participant stated:

The child can choke and die. (Participant 9)

Multiple participants reported that community members believe healthcare workers want to harm children with nasogastric tubes. One participant shared religious views she heard in the community about healthcare workers wanting to kill children with nasogastric tubes:

People in the community do not have the knowledge of what happens in the health facilities. Some people would say that the health care workers want to help in depopulating the country by having more children dead because by putting the tube in the nose of the child, it can block the nostrils making it difficult for the child to breathe. Some may think that it is the works of the devil because a child cannot survive the tube and they may say that it is satanic. (Participant 10)

#### Beliefs about breastfeeding

All participants shared something positive about breastfeeding and its effects on their child. The most common comments were that breastfeeding supports their child’s health and promotes growth:

They said we should breastfeed the child often so that their body can be healthy and strong. (Participant 8)

Several participants mentioned that breast milk provides nutrients for their child:

My understanding is that breast milk has all the nutrients for the child especially the colostrum. (Participant 7)

One participant discussed how breastfeeding improves the bond between mothers and their children:

Breastfeeding also enhances love and bond between the child and the mother. (Participant 6)

Several mothers were advised that they should exclusively breastfeed after delivery for some amount of time:

They used to tell us to exclusively breastfeed the child for six months so that s/he should be healthy and be protected from diseases*.* (Participant 5)

Three women mentioned that their child’s health would suffer in some way if they do not breastfeed:

The child’s health depends on breast milk and if you stop breastfeeding, the child’s health can be compromised. (Participant 3)

The most common sources of information about breastfeeding that participants cited were the antenatal clinic, the antenatal hospital ward or the children’s clinic.

## Discussion

This study highlights caregiver perceptions and experiences of hospital feeding practices for infants admitted with severe pneumonia to a government tertiary referral hospital in Malawi. While mothers generally understood that some children with pneumonia may experience feeding difficulties and aspiration risk, they expressed substantial concerns about discontinuing breastfeeding and instead feeding their infants through a nasogastric tube. These concerns were present regardless of whether a medical provider recommended temporary cessation of direct breastfeeding due to aspiration risk. Alarmingly, we also found that many communities perceive nasogastric tubes to be harmful and believe that medical providers who recommend feeding tubes have malicious intent and aim to harm, or even kill, children. These troubling findings have serious implications for the outcomes of children hospitalised with pneumonia.

Our study is one of the few to evaluate caregiver feelings about nasogastric tube feeding in children in Africa. A study conducted at tertiary hospitals in Nigeria showed that most caregivers accepted nasogastric tube feeding for their children, while those that declined did so because they thought the tubes would occlude the child’s airway.^14^ These caregiver concerns from Nigeria are similar to our findings in Malawi that mothers worry nasogastric tubes cause children to ‘choke’. While we did not directly ask mothers if they refused nasogastric tube insertion and/or feeding, we suspect that a few declined, as only five reported they were told to stop breastfeeding. However, seven infants required bubble CPAP non-invasive ventilation, and KCH policy requires infants on bubble CPAP to be fed breast milk by nasogastric tube only. Focus group discussions with mothers of children enrolled in a bubble CPAP trial in Malawi showed that mothers had widely negative perceptions of oxygen tubes, with some even reporting they intermittently removed them from their children.^4^ Although not discussed in the focus groups, the authors thought that based on these findings, mothers were likely to have either removed nasogastric tubes or kept them in place but not used them for feeding their children. It is unsurprising that mothers in our study expressed significant concerns about nasogastric tube feeding, particularly given that many had heard from community members that nasogastric tubes cause children to ‘choke’ or even die. Furthermore, some mothers who were not advised to discontinue breastfeeding expressed uncertainty about nasogastric tube feeding, as they did not realise that expressed breast milk could be administered via the tube.

We also found that all mothers had knowledge about the health benefits of breastfeeding. Benefits commonly cited were provision of nutrients and improvement of bonding between mothers and infants. This positive finding in Malawian mothers echoes other studies in Africa showing that mothers understand that breastfeeding is important for children’s health and increases bonding.^15 16^ A study in South Africa on mothers’ perceptions of infant feeding practices found mothers believe that unwillingness to eat is a sign of ill health.^17^ Considering the extensive view that feeding is a surrogate marker of health, in combination with the strong efforts to promote exclusive breastfeeding in Africa, it is unsurprising that mothers in our study had concerns about not allowing their infants to breastfeed. Additionally, it was apparent that some mothers did not understand that instead of breastfeeding, mothers can express breast milk and feed it to their infants through the nasogastric tube. Some mothers who were told to stop breastfeeding were more accepting of nasogastric tube feeding after they understood their infant would still be receiving their expressed breast milk. This suggests that with improved understanding of the benefits of tube feeding during severe respiratory illness, including provision of consistent nutrition, safety in comparison with oral feeding, and promotion of continued breast milk production after recovery, mothers may be more receptive to this feeding practice.

The concerning caregiver perception that nasogastric tube feeding is harmful has critical implications for the outcomes of hospitalised children with pneumonia. These negative perceptions may lead to the refusal or premature removal of nasogastric tubes from children, resulting in catastrophic aspiration events during feeding, as may have been observed in the bubble CPAP trial in Malawi.^4^ Mothers may also be less likely to promptly seek care for their children out of fear that nasogastric tubes are dangerous, as seen in previous studies on perceptions of oxygen, leading to more severe illnesses on presentation to care.^6^ Aspiration events during feeding can be devastating and lead to increased mortality in children with pneumonia. To safely treat children with pneumonia with low-flow oxygen and emerging technologies like bubble CPAP, it is essential to address caregiver concerns about nasogastric tubes and provide comprehensive education on the importance of their temporary use in the hospital. This may involve standardised training for medical providers on educating caregivers about the use of nasogastric tubes in hospitalised children. We must also validate the benefits of breastfeeding while emphasising that breast milk can still be safely administered through nasogastric tubes to support infant nutrition during hospitalisation. Community education initiatives led by community health workers may also be an effective strategy to disseminate this information to caregivers.^18^

There are several notable limitations to this study. First, the opinions and views of the mothers interviewed may not represent the views of all mothers in Malawi and in other settings in Africa. For instance, our study was conducted at a single hospital in the Central Region of Malawi. Also, mothers who never presented to the hospital were not included in the interviews. Despite this sampling bias, mothers were able to share community and family member views, and the sample size of mothers included was adequate to achieve thematic saturation. Second, mothers may have been inclined to provide more favourable answers or responses they felt would be desired by the research team. To minimise this social desirability bias, we used experienced female interviewers from Malawi who were not medical providers and were trained to ask questions without judgement. Third, we did not interview the medical providers at KCH to understand their experiences with and perspectives on recommending nasogastric tube feeding for hospitalised children. The beliefs of medical providers may have influenced the education they provided to mothers regarding nasogastric tube feeding, including the possibility that they did not recommend the use of nasogastric tubes, thereby affecting mothers’ perspectives on nasogastric tube feeding. A key next step will be to similarly assess medical provider perspectives on nasogastric tube feeding. Despite these limitations, our study offers unique insights into caregiver perceptions and experiences of hospital feeding practices for infants with pneumonia in Malawi.

## Conclusion

In Malawi, there are widespread concerns about hospital feeding practices for sick children, with mothers and community members often citing that nasogastric tubes are harmful or kill children and that healthcare providers who recommend nasogastric tube feeding may have malicious intent. When nasogastric tube feeding is recommended, mothers have additional worries about not breastfeeding their infants, which may further result in non-adherence to medical therapies. These findings have implications for the outcomes of children hospitalised with pneumonia in LMICs like Malawi. Contextually appropriate caregiver and community education is urgently needed to improve the acceptance of hospital feeding practices.

## Supplementary material

10.1136/bmjopen-2024-094793 online supplemental file 1

10.1136/bmjopen-2024-094793 online supplemental file 2

## Data Availability

Data are available upon reasonable request.
