# Supplementary material for: Perceptions of hospital feeding practices among mothers of infants with severe pneumonia in Malawi: a qualitative descriptive study
Source: BMJ Open. 2025 Jun 8;15(6):e094793. doi: 10.1136/bmjopen-2024-094793 (PMC12161392; doi:10.1136/bmjopen-2024-094793)
Supplement: online supplemental file 1 [file bmjopen-15-6-s001.pdf]

Patient ID: ______
Date (DD/MM/YYYY): ________
Initials of facilitator: _______
Mother ID: _________

Thank you for joining this interview. You have been invited to participate in this interview because your child is enrolled in a research study while they are hospitalized. We want to better understand your experiences during your child's hospitalization.

1. How long has your child been sick?
    a. How was your journey to the hospital?
    b. Where is home for you?

2. Why has your child been admitted to the hospital?
    a. What is your child suffering from?
    b. How long have you been admitted to the hospital?

3. Had you heard about "difficult breathing" or "pneumonia" before your child was hospitalized?
    If YES:
    a. Please share what you had heard and who you heard it from.
    b. What problems do children with "difficult breathing" or "pneumonia" have?
    c. Had you heard that children who have "difficult breathing" or "pneumonia" may have trouble feeding?

4. During the hospitalization, were you told that your child should stop breastfeeding?
    IF YES:
    a. Did someone from the medical team explain why this is important for your child's health?
    b. What is your understanding of why some children are not allowed to breastfeed when they have "difficult breathing" or "pneumonia"?
    c. Can you describe what it felt like to not allow your child to breastfeed?
    d. Do you have any concerns that withholding breastfeeding can harm your child? Explain.
    e. Did the medical team recommend that you feed your child with a nasogastric tube (NGT)?
    f. What do you know about feeding children with NGTs?
    g. Do you have any concerns that NGT feeds are harmful to children? Explain.
    h. What are the perceptions in your community on feeding children with NGTs?
    i. How did these perceptions affect your decision about feeding with an NGT?
    j. Did you feed your child with an NGT?
    k. Did you breastfeed your child while they had a NGT in place?

Patient ID: ______
Date (DD/MM/YYYY): ________
Initials of facilitator: _______
Mother ID: _________

IF NO:

l. What is your understanding of why some children are not allowed to breastfeed when they have "difficult breathing" or "pneumonia"?
m. Do you feel that your child should stop breastfeeding? Explain.
n. What do you know about feeding children with a nasogastric tube (NGT)?
o. Do you have any concerns that NGT feeds are harmful to children? Explain.
p. What are the perceptions in your community on feeding children with NGT feeds?
q. Do you feel that your child should have an NGT for feeding?

5. What is your understanding of breastfeeding and how it relates to your child's health?
    a. Who have you learned about breastfeeding from?

Thank you for your participation in this interview today. This information will help us design future educational materials to help providers talk to mothers and caregivers about treatment for "difficult breathing" or "pneumonia". Please let us know if you have any questions.
