# Supplementary material for: Perceptions of hospital feeding practices among mothers of infants with severe pneumonia in Malawi: a qualitative descriptive study
Source: BMJ Open. 2025 Jun 8;15(6):e094793. doi: 10.1136/bmjopen-2024-094793 (PMC12161392; doi:10.1136/bmjopen-2024-094793)
Supplement: online supplemental file 2 [file bmjopen-15-6-s002.pdf]

Patient ID: ______
Date (DD/MM/YYYY): ________
Initials of facilitator: _______
Mother ID: _________

Zikomo pokhala nawo mu zokambirana izi. Mwayitanidwa kuti mutenge nawo mbali mu zokambirana izi chifukwa chakuti mwana wanu akutenga mbali mu kafukufuku nthawi imene anali mu chipatala. Tikufuna kumvetsetsa zimene mwakumana nazo nthawi imene mwana wanu ali mchipatala.

1. Mwana wanu wadwala nthawi yayitali bwanji?
    a. Ulendo wanu obwera ku chipatala unali otani?
    b. Kwanu ndi kuti?
2. Mwana wanu wagonekedwa mchipatala chifukwa chani?
    a. Mwana wanu akudwala chani?
    b. Mwagonekedwa mu chipatala kwa nthawi yayitali bwanji?
3. Munali munvako za "kuvutika kupuma" kapena "chibayo" mwana wanu asanagonekedwe?
   NGATI EYA:
    a. Chonde fotokozani zimene munanva komanso kumene munazinva.
    b. Ana amene "akuvutika kupuma" kapena ali ndi "chibayo" amakhala ndi mavuto anji?
    c. Munali mutamvako kuti ana amene amavutika kupuma chifukwa cha chibayo akhoza kukhala ndi mavuto kudya?
4. Nthawi imene mumagonekedwa mchipatala, munawuzidwa kuti mwana wanu asiye kuyamwa?
   NGATI EYA:
    a. M'modzi wa ma dokotala anakulongosolerani kufunika kwa zimenezi ku thanzi la mwana wanu?
    b. kumvetsetsa kwanu ndi kotani kwa zifukwa zimene ana ena samaloledwa kuyamwa akakhala kuti "akuvutika kupuma" kapena "ali ndi zibayo"?
    c. Mungathe kufotokoza m'mene munanvera pomukaniza mwana wanu kuyamwa?
    d. Muli ndi nkhawa iliyonse kuti kukaniza mwana wanu kuyamwa kukhoza kumuonga mwana wanu? Longosolani.
    e. Madokotala anakuwuzani kuti muzimudyetsa mwana wanu ndi chubu chodzera m'phuno (NGT)?
    f. Mukudziwa chani zokhudza kudyetsa ana ndi NGT?
    g. Muli ndi nkhawa iliyonse kuti kudyetsa kudzera mma chubu oyikidwa m'phuno kuli ndi chiophyezo kwa ana? Longosolani
    h. Anthu a m'dera amaganiza chani zokhudza kudyetsa ana kudzera mu chubu yoyikidwa m'phuno?
    i. Maganizo amenewa anakhudza bwanji chiganizo chanu chokhudza kudyetsa ana kudzera mu chubu yoyikidwa m'mphuno?
    j. Munadyetsa mwana wanu ndu chubu choyikidwa mphuno?

Patient ID: ______
Date (DD/MM/YYYY): ________
Initials of facilitator: _______
Mother ID: _________

k. Munayamwitsa mwana wanu ali ndi chubu choyikidwa m'mphuno?
NGATI AYI:
l. Kumvetsetsa kwanu kwa zifukwa zimene ana ena samaloledwa kuti ayamwe pamene "akuvutika kupuma" kapena "ali ndi zibayo" ndi Kotani?
m. Mukuona kuti mwana wanu akuyenera asiye kuyamwa? Longosolani.
n. Mukudziwapo chani zokhudza kudyetsa ana kudzera mu chubu yoyikidwa m'mphuno?
o. Muli ndi nkhawa iliyonse kuti kudyetsa kudzera mu chubu yoyikidwa m'phuno kuli ndi chiophyezo kwa ana? Longosolani
p. Anthu a m'dera amaganiza chani zokhudza kudyetsa ana kudzera mu chubu yoyikidwa m'phuno?
q. Mukuganiza kuti mwana wanu akuyenera akhale ndi chubu yoyikidwa m'mphuno kuti azidyera m'menemo?

5. Kumvetsetsa kwanu kwa kuyamwitsa komanso mmene kumakhudzira thanzi la mwana wanu ndi Kotani?
   a. Mwaphunzira za kuyamwitsa kuchokera kwa ndani?

Zikomo chifukwa cha kutenga mbali kwanu mu zokambiranazi lero. Uthenga uwu utithandiza kukonza zida zophunzirira za mtsogolo zoti zizathandize opereka chithandizo poyankhula ndi azimayi komanso opereka chisamaliro zokhudza chithandizo la "uvutika kupuma" kapena "chibayo". Chonde ndiuzeni ngati muli ndi mafunso ena aliwonse.
