# Supplementary material for: Perceptions of hospital feeding practices among mothers of infants with severe pneumonia in Malawi: a qualitative descriptive study
Source: BMJ Open. 2025 Jun 8;15(6):e094793. doi: 10.1136/bmjopen-2024-094793 (PMC12161392; doi:10.1136/bmjopen-2024-094793)
Supplement: Supplementary file 3 [file bmjopen-2024-094793.reviewer_comments.pdf]

# PEER REVIEW HISTORY

BMJ Open publishes all reviews undertaken for accepted manuscripts. Reviewers are asked to complete a checklist review form and are provided with free text boxes to elaborate on their assessment. These free text comments are reproduced below.

## ARTICLE DETAILS

### Title (Provisional)

Perceptions of hospital feeding practices among mothers of infants with severe pneumonia in Malawi: A qualitative descriptive study

### Authors

Hoekstra, Nadia E.; Craven, Dalton M; Tsidya, Mercy; Thom, Annie; Bula, Agatha; van der Zalm, Marieke; Mvalo, Tisungane; McCollum, Eric D.

---

## VERSION 1 - REVIEW

---

| | |
|---|---|
| **Reviewer** | **1** |
| **Name** | **Toh, Teck-Hock** |
| **Affiliation** | **Sibu Hospital** |
| **Date** | **01-Apr-2025** |
| **COI** | **None** |

---

Title: Perceptions of hospital feeding practices among mothers of infants with pneumonia in Malawi: A qualitative descriptive study

Thank you for your invaluable contribution to a critical topic of significant importance to a country like Malawi. The work is instrumental in shaping the discourse on infant nutrition, and the effort put into it is much appreciated. However, I have some personal views and concerns that I believe could further enhance the manuscript:

1. Lack of discussion on WHO recommendations

The paper does not explicitly mention the WHO guidelines in the introduction and discussion, which recommend exclusive breastfeeding for six months and continued breastfeeding for up to two years. This omission weakens the study's discussion on best practices for infant nutrition. In addition, advising mothers to stop breastfeeding without a valid reason and proper support is a major ethical concern.

2. NG feeding practices not specified.

While the paper acknowledges the risk of aspiration for children with pneumonia and tachypnoea, it lacks clarity on the standard practice followed. It should specify whether expressed breast milk (EBM) with NG feeding was the preferred alternative and if mothers

were supported in maintaining lactation. This confusion was reflected by Participant 12 on page 17, causing hesitancy in the mother to "stop" breastfeeding and allow NG feeding using EBM. The absence of this detail leaves a significant gap in understanding hospital practices, and your clarification on this matter would significantly enhance the study's comprehensiveness.

3. Context of NG feeding in pneumonia cases

It's important to note that not all infants with pneumonia require NG feeding. The paper should consider this when analysing mothers' perspectives. It would strengthen the discussion to differentiate between levels of illness severity and how feeding recommendations vary. The study commendably highlights the dedication of Malawian mothers to breastfeeding, contrasting with challenges in other countries. This is an important positive aspect that should be emphasised in the discussion. The manuscript (and perhaps the medical practice) was not clear about the alternative feedings (EBM vs formula via NG) when the child is tachypnoeic; I would believe it explains why the overwhelming concern (and dislike/disagreement) of the breastfeeding mother to "stop" breastfeed when advised.

4. Incorrect use of "sucking" instead of "suckling"

Throughout the paper, "suckling" should be used instead of "sucking," as it is the correct term for breastfeeding technique. A baby sucks on the bottle, but suckling is a natural, instinctive action associated with breastfeeding to draw milk from the nipple. Sucking is a broader term that means applying suction but does not necessarily imply feeding.

5. Sample size

In qualitative research, the adequacy of a sample size depends on data saturation, meaning that no new themes or insights emerge from additional participants. In this paper, though the research question is narrow and focussed (i.e. exploring mothers' experience with breastfeeding during infants' pneumonia), the sample lacks diversity in experiences (e.g., different severities of illness, different feeding practices details, e.g., duration, age of the infant, the introduction of solids, etc.). The study does not explicitly mention whether saturation was achieved (hence, unsure if 14 participants were sufficient).

6. Lack of clarity on participant backgrounds to help interpret the findings

Similar to (5), the study includes 14 participants, but essential details such as their infants' illness severity, infant age (and hence breastfeeding duration, and introduction of solids) are not presented. This context is crucial for interpreting the findings.

7. English language and grammar

The paper should maintain consistency in British English. Additionally, minor grammar issues should be addressed for clarity and professionalism.

| | |
|---|---|
| **Reviewer** | **2** |
| **Name** | **Pajuelo, Mónica** |
| **Affiliation** | **Universidad Peruana Cayetano Heredia, Cellular and Molecular Sciences** |
| **Date** | **12-Apr-2025** |
| **COI** | **None** |

The authors conducted a qualitative study aimed at exploring mothers' perceptions of feeding practices in children hospitalized for pneumonia treatment. The results are interesting and important to document in this setting in order to try some strategies to inform to the population.

Some suggestions:

Abstract: It is recommended to include the specific time period of the study for better contextualization.

Introduction: This section is clear.

Methods: To enhance clarity, provide details about the interview guidelines and the process of recruiting mothers. and include the study period (e.g., March 15 to April 7).

Specify the reason for conducting 14 interviews. While the study is qualitative, reporting an approximate number of children admitted during this time frame could add valuable context. Address whether any mothers declined to participate and whether a pilot interview was conducted. Additionally, elaborate on the training and occupations of the local experts involved to highlight methodological rigor.

Results: In table 1. Consider using the median rather than the mean to describe the number of children. Given the limited information presented in the table, integrating these results directly into the text could improve the overall narrative and make findings more accessible.

## VERSION 1 - AUTHOR RESPONSE

**Reviewer: 1**
Dr. Teck-Hock Toh, Sibu Hospital
Comments to the Author:
Title: Perceptions of hospital feeding practices among mothers of infants with pneumonia in Malawi: A qualitative descriptive study

Thank you for your invaluable contribution to a critical topic of significant importance to a country like Malawi. The work is instrumental in shaping the discourse on infant nutrition, and the effort put into it is much appreciated.

However, I have some personal views and concerns that I believe could further enhance the manuscript:

1. **Reviewer 1:** Lack of discussion on WHO recommendations
   The paper does not explicitly mention the WHO guidelines in the introduction and discussion, which recommend exclusive breastfeeding for six months and continued breastfeeding for up to two years. This omission weakens the study's discussion on best practices for infant nutrition. In addition, advising mothers to stop breastfeeding without a valid reason and proper support is a major ethical concern.

   **Author's response:** Thank you for your insightful comment. We have now incorporated the WHO nutrition recommendations into the background section of our manuscript to explicitly reference best practices for infant feeding. The revised section emphasizes that WHO guidelines recommend exclusive breastfeeding for the first six months of life and continued breastfeeding up to two years.

   Additionally, we have clarified that temporary cessation of oral feeding is considered only in infants hospitalized with severe pneumonia who are experiencing respiratory distress, particularly those on non-invasive ventilation. These infants are at high risk of aspiration, which can lead to life-threatening events. This precautionary approach is aligned with common clinical practices for managing feeding in critically ill infants. We acknowledge the ethical concerns associated with advising mothers to stop breastfeeding without appropriate medical justification and support.

   The revised section of the background is as follows:

   "Some pneumonia-related deaths in LMICs may be attributed to aspiration during oral feeding [4]. Children experiencing respiratory distress from pneumonia have an increased risk of aspiration from swallow dysfunction [5]. Emerging treatments for pneumonia in low-resource settings, such as non-invasive ventilation with continuous positive airway pressure (CPAP) [6], may further contribute to swallowing difficulties and aspiration risk during feeding [7,8]. To reduce the risk of life-threatening aspiration events in children hospitalized with severe respiratory distress, particularly those requiring non-invasive ventilation, medical providers may temporarily recommend nasogastric tube feedings rather than oral feeding [9]. Nasogastric tubes are narrow, plastic tubes inserted through the nose, down the throat and esophagus, into the stomach, and used to deliver liquid nutrition and medications directly into the stomach.

   Current World Health Organization (WHO) guidelines on child nutrition recommend exclusive, on-demand breastfeeding for infants during the first six months of life and continued breastfeeding up to two years [10]. For emerging pneumonia treatments to be safe, feasible, and effective, ensuring caregiver acceptance and adherence to temporary alternative hospital feeding practices is essential."

2. **Reviewer 1:** NG feeding practices not specified.
   While the paper acknowledges the risk of aspiration for children with pneumonia and tachypnoea, it lacks clarity on the standard practice followed. It should specify whether expressed breast milk (EBM) with NG feeding was the preferred alternative and if mothers were supported in maintaining lactation. This confusion was reflected by Participant 12 on page 17, causing hesitancy in the mother to "stop" breastfeeding and allow NG feeding using EBM. The absence of this detail leaves a significant gap in understanding hospital practices, and your clarification on this matter would significantly enhance the study's comprehensiveness.

   **Author's response:** Thank you for this comment. We agree that further clarification is needed regarding nasogastric feeding practices. We have now explicitly stated in the Methods section that mothers who are breastfeeding were instructed to express breast milk and provider it to their children through a nasogastric tube. The changes are as follows:

   "At KCH, infants and children who have severe respiratory distress and require bubble CPAP non-invasive ventilation are not permitted to feed orally. As a result, breastfeeding mothers are instructed by medical providers to cease direct breastfeeding. Instead, a nasogastric tube is inserted to facilitate feeding. Mothers are advised to express breast milk and administer it through the nasogastric tube, as formula is not available in this setting."

   We also clarified in the Results section that mothers who were advised to stop direct breastfeeding due to aspiration risk were instructed to express breast milk and feed it to their infant via a nasogastric tube. The revised text reads:

   "Five mothers (35.7%) reported being advised by a medical provider to discontinue direct breastfeeding. In these cases, the providers recommended insertion of a nasogastric tube and feeding with expressed breast milk via the tube."

   Furthermore, we found that before receiving explanations from medical providers, some mothers were unaware that expressed breast milk could be administered via nasogastric tube. This lack of clarity contributed to hesitancy, as reflected in Participant 12's response. We have refined our explanation of this quote to highlight the misconceptions surrounding nasogastric feeding and lactation maintenance. Additionally, some mothers who were not advised to stop breastfeeding did not realize that infants with nasogastric tubes could receive expressed breast milk.

   "Before receiving an explanation from a medical provider, this participant initially assumed her child would receive formula through a nasogastric tube instead of breastfeeding.

*“My only concern was that when my child will resume breastfeeding, the breast milk will be sour or not user friendly because I would stay for long without him breast feeding. I also thought that my breasts will swell because I thought we were to use formula but then they said we were to express the breast milk and pour in the NGT so I was okay with that.”* (Participant 12)”

3. **Reviewer 1:** Context of NG feeding in pneumonia cases
   It's important to note that not all infants with pneumonia require NG feeding. The paper should consider this when analysing mothers' perspectives. It would strengthen the discussion to differentiate between levels of illness severity and how feeding recommendations vary.

   **Author’s response:** Thank you for this important comment. We recognize the need to clarify that not all infants with pneumonia require nasogastric feeding. In our study, infants with severe pneumonia required various levels of respiratory support, including bubble CPAP, low flow oxygen, or no respiratory support. To ensure representation across illness severity, we intentionally interviewed mothers whose infants required bubble CPAP as well as those whose infants did not. We have clarified this distinction in the Methods section, as follows:

   “Mothers of infants admitted to the pediatric ward at KCH who were participating in the parent observational study were chosen for interviews through opportunistic and convenience sampling. Specifically, mothers who were present at their infant’s bedside during the study period were eligible to participate. We approached mothers of infants who required bubble CPAP non-invasive ventilation and those whose infants received conventional low flow oxygen or no respiratory support. Infant age, sex, and bubble CPAP use during hospitalization were documented, alongside maternal demographic data, including age, number of children, and highest education level achieved.”

   Additionally, only five mothers (35.7%) reported that medical providers recommended stopping direct breastfeeding. Infants on bubble CPAP and those with severe respiratory distress are typically restricted from oral feeding at Kamuzu Central Hospital due to the increased aspiration risk. We have clarified this point in the Discussion, as follows:

   “This study highlights caregiver perceptions and experiences of hospital feeding practices for infants with severe pneumonia admitted to a government tertiary referral hospital in Malawi. While mothers generally understand that some children with pneumonia may experience feeding difficulties and aspiration risk, they expressed substantial concerns about discontinuing breastfeeding and instead feeding their infants through a nasogastric tube. These concerns were present regardless of whether a medical provider recommended temporary cessation of direct breastfeeding due to aspiration risk. Alarmingly, we also found that many communities perceive nasogastric tubes to be harmful and believe that

medical providers who recommend feeding tubes have malicious intent and aim to harm, or even kill, children. These troubling findings have serious implications for the outcomes of children hospitalized with pneumonia."

4. **Reviewer 1:** The study commendably highlights the dedication of Malawian mothers to breastfeeding, contrasting with challenges in other countries. This is an important positive aspect that should be emphasised in the discussion.

   **Author's response:** Thank you for this insightful comment. We agree that the strong commitment of Malawian mothers to breastfeeding is an important positive aspect of our findings. We have emphasized this in the discussion section, highlighting that all mothers demonstrated knowledge of the health benefits of breastfeeding, including its role in providing essential nutrients and strengthening the mother-infant bond. This finding aligns with existing studies in Africa, which consistently show that mothers recognize breastfeeding as critical to child health and development. Our changes to the Discussion are as follows:

   "We also found that all mothers have knowledge about the health benefits of breastfeeding. Benefits commonly cited were provision of nutrients and improvement of bonding between mothers and infants. This positive finding in Malawian mothers echoes other studies in Africa showing that mothers understand that breastfeeding is important for children's health and increases bonding [15, 16]."

5. **Reviewer 1:** The manuscript (and perhaps the medical practice) was not clear about the alternative feedings (EBM vs formula via NG) when the child is tachypnoeic; I would believe it explains why the overwhelming concern (and dislike/disagreement) of the breastfeeding mother to "stop" breastfeed when advised.

   **Author's response:**
   Thank you for this comment. We recognize the need to clarify that mothers who were advised to temporarily stop direct breastfeeding due to aspiration risk were instructed to express breast milk and feed it to their infant via a nasogastric tube. This has been explicitly stated in the Methods section, as follows:

   "At KCH, infants and children who have severe respiratory distress and require bubble CPAP non-invasive ventilation are not permitted to feed orally. As a result, breastfeeding mothers are instructed by medical providers to cease direct breastfeeding. Instead, a nasogastric tube is inserted to facilitate feeding. Mothers are advised to express breast milk and administer it through the nasogastric tube, as formula is not available in this setting."

   We have also stated it in the Results section as follows:

“Five mothers (35.7%) reported being advised by a medical provider to discontinue direct breastfeeding. In these cases, the providers recommended insertion of a nasogastric tube and feeding with expressed breast milk via the tube.”

Additionally, we acknowledge that some mothers who were not advised to stop breastfeeding- but had heard about or observed nasogastric tube feeding- were unaware that expressed breast milk could still be provided through the tube. This knowledge gap contributed to concerns and hesitancy surrounding nasogastric feeding. To address this, we have revised the Discussion, as follows:

“It is unsurprising that mothers in our study expressed significant concerns about nasogastric tube feeding, particularly given that many had heard from community members that nasogastric tubes cause children to choke or even die. Furthermore, some mothers who were not advised to discontinue breastfeeding expressed uncertainty about nasogastric feeding, as they did not realize that expressed breast milk could be administered via the tube.”

In the Discussion we also mention that it is important for medical providers to explain that breast milk can be given through nasogastric tubes, as follows:

“We must also validate the benefits of breastfeeding while emphasizing that breast milk can still be safely administered through nasogastric tubes to support infant nutrition during hospitalization.”

6. **Reviewer 1:** Incorrect use of "sucking" instead of "suckling"
   Throughout the paper, "suckling" should be used instead of "sucking," as it is the correct term for breastfeeding technique. A baby sucks on the bottle, but suckling is a natural, instinctive action associated with breastfeeding to draw milk from the nipple. Sucking is a broader term that means applying suction but does not necessarily imply feeding.

   **Author’s response:** Thank you for bringing this to our attention. We recognize that “suckling” is technically the correct word for breastfeeding, as it describes the natural, instinctive action of drawing milk from the nipple. However, the term “sucking” was used directly in a quote from a mother during the interview. To maintain the integrity of participant responses, we have retained “sucking” in the quoted text to accurately reflect the mother’s own words. We have not used the term “sucking” elsewhere in the manuscript.

7. **Reviewer 1:** Sample size
   In qualitative research, the adequacy of a sample size depends on data saturation, meaning that no new themes or insights emerge from additional participants. In this paper, though the research question is

narrow and focused (i.e. exploring mothers' experience with breastfeeding during infants' pneumonia), the sample lacks diversity in experiences (e.g., different severities of illness, different feeding practices details, e.g., duration, age of the infant, the introduction of solids, etc.). The study does not explicitly mention whether saturation was achieved (hence, unsure if 14 participants were sufficient).

**Author's response:** Thank you for this helpful comment. We recognize the important of providing additional context to ensure clarify regarding sample diversity and data saturation. To improve transparency, we have added infant age data to Table 1. Additionally, we have clarified in the Methods section that all enrolled infants met the World Health Organization criteria for "severe pneumonia" and, therefore, had severe disease. Within this group, infants required varying levels of respiratory support including bubble CPAP, low-flow oxygen, or no respiratory support. To ensure representation across illness severity, we intentionally interviewed mothers whose infants required CPAP and those whose infants did not. This distinction is now explicitly stated in the Methods section.

Infants experiencing respiratory distress and requiring CPAP are typically at the highest risk of aspiration, prompting providers to recommend withholding oral feeds. However, due to hospital resource limitations, CPAP availability varies, and some infants experiencing respiratory distress may not receive CPAP yet may have oral feeds withheld.

Furthermore, all infants in this study were breastfed. While we did not document whether they were also receiving solid food, we have clarified their breastfeeding status in the Methods section as follows:

"We conducted in-depth interviews with 14 mothers of breastfed infants 0- to 12-months of age who were admitted to the pediatric ward with WHO-defined [12] and enrolled in an observational study that evaluated feeding and swallowing in breastfed infants with severe pneumonia."

To better explain the thematic analysis approach and explicitly confirm data saturation, we have revised the Methods section as follows:

"An initial codebook was developed from the interview guide using pre-defined themes. Following each interview, members of the research team read the transcript in full and met to discuss emerging themes. Codes were added to the codebook iteratively, and
the final version was established through consensus by the research team. Each transcript was then coded by two of three independent reviewers (NEH, DMC and MT). The team met weekly to review and reconcile all areas of discrepancy until agreement of the coded text was reached via consensus.

The team identified themes and subthemes, then used Microsoft Excel to construct matrices summarizing them. All themes were saturated after 14 interviews."

8. **Reviewer 1:** Lack of clarity on participant backgrounds to help interpret the findings. Similar to (5), the study includes 14 participants, but essential details such as their infants' illness severity, infant age (and hence breastfeeding duration, and introduction of solids) are not presented. This context is crucial for interpreting the findings.

   **Author's response:** We agree with this comment. We recognize that providing additional details on participant backgrounds, including infant illness severity and age, is important for contextualizing findings.

   To improve transparency, we have added infant age, sex, and bubble CPAP data to Table 1. Additionally, we have clarified in the Methods section that all enrolled infants met the World Health Organization criteria for "severe pneumonia" and, therefore, had severe disease. Within this group, infants required varying levels of respiratory support including bubble CPAP, low-flow oxygen, or no respiratory support. To ensure representation across illness severity, we intentionally interviewed mothers whose infants required CPAP and those whose infants did not.

   Furthermore, all infants in this study were breastfed. While we did not document whether they were also receiving solid food, we have clarified their breastfeeding status in the Methods section as follows:

   "We conducted in-depth interviews with 14 mothers of breastfed infants 0- to 12-months of age who were admitted to the pediatric ward with WHO-defined [12] and enrolled in an observational study that evaluated feeding and swallowing in breastfed infants with severe pneumonia."

9. **Reviewer 1:** English language and grammar
   The paper should maintain consistency in British English. Additionally, minor grammar issues should be addressed for clarity and professionalism.

   **Author's response:** Thank you for identifying grammatical inconsistencies. We have carefully reviewed the manuscript to correct minor grammatical errors and improve overall clarity. Regarding the use of British English, we defer to the editor's preference and are happy to adjust accordingly based on journal guidelines.

**Reviewer: 2**
Dr. Mónica Pajuelo, Universidad Peruana Cayetano Heredia, Johns Hopkins University

The authors conducted a qualitative study aimed at exploring mothers' perceptions of feeding practices in children hospitalized for pneumonia treatment. The results are interesting and important to document in this setting in order to try some strategies to inform to the population.

1. **Reviewer 2:** Abstract: It is recommended to include the specific time period of the study for better contextualization.

   **Author's response:** Thank you for this recommendation. We have revised the abstract to include the time period of the study to improve contextualization and ensure clarity for readers.

2. **Reviewer 2:** Introduction: This section is clear.

   **Author's response:** Thank you for your feedback. We appreciate your positive assessment of the introduction.

3. **Reviewer 2:** Methods: To enhance clarity, provide details about the interview guidelines and the process of recruiting mothers. and include the study period (e.g., March 15 to April 7).

   **Author's response:** Thank you for your suggestion. We have attached the interview guide as a supplementary file for reference. Additionally, we have clarified the process of recruiting mothers and included the specific study period in the Methods section, as follows:

   "Mothers of infants admitted to the pediatric ward at KCH who were participating in the parent observational study were chosen for interviews through opportunistic and convenience sampling. Specifically, mothers who were present at their infant's bedside during the study period were eligible to participate. We approached mothers of infants who required bubble CPAP non-invasive ventilation and those whose infants received conventional low flow oxygen or no respiratory support. Infant age, sex, and bubble CPAP use during hospitalization were documented, alongside maternal demographic data, including age, number of children, and highest education level achieved."

   We have also included the specific study period in the Methods, as follows:

   "This descriptive qualitative study was conducted at KCH from March 28 through April 19, 2023."

4. **Reviewer 2:** Specify the reason for conducting 14 interviews. While the study is qualitative, reporting an approximate number of children admitted during this time frame could add valuable context.

**Author's response:** Thank you for this comment. We conducted 14 interviews based on the principle of thematic saturation, where no new themes emerged after this number of interviews. The mothers were selected from an ongoing observational study evaluating feeding and swallowing in breastfed infants with severe pneumonia. We used opportunistic and convenience sampling, approaching mothers who were present at their infant's bedside during the study period. While our study was qualitative and not designed to quantify the total number of infants during this time, we acknowledge that providing an estimate of total admissions could add broader context. The study was conducted at Kamuzu Central Hospital, where approximately 40 infants aged 0-5 months are admitted with severe pneumonia per month on average. We have revised the methods section to include this information, as below:

"Kamuzu Central Hospital (KCH), located in Lilongwe, Malawi, is a tertiary referral hospital serving the Central Region of the country. On average, approximately 40 infants aged 0-5 months are admitted to KCH each month with severe pneumonia [3]."

5. **Reviewer 2:** Address whether any mothers declined to participate and whether a pilot interview was conducted.

   **Author's response:** Thank you for this helpful comment. We did pilot the interview material prior to conducting interviews with mothers. Two mothers declined to participate. We have added this information to the Methods and Results sections, as follows:

   "Interviews were held in a private room at KCH and conducted in the local language of Chichewa by a facilitator trained in qualitative research. The interview guide was developed by the research team, reviewed by local experts, and piloted prior to interviews (Supplementary File)."

   "A total of 14 mothers participated in interviews, while two declined. Table 1 reports the demographic characteristics of the participating mothers and their infants with severe pneumonia."

6. **Reviewer 2:** Additionally, elaborate on the training and occupations of the local experts involved to highlight methodological rigor.

   **Author's response:** Data was collected by two experienced qualitative researchers working at the University of North Carolina (UNC) Project Malawi. UNC Project Malawi has a qualitative team that supports all qualitative studies conducted at UNC Project Malawi. The researchers have been formally trained in qualitative research methodologies, data collection, and data analysis techniques. Each has over 8 years of experience in conducting qualitative research studies. Prior to data collection, they participated in a two-day protocol orientation to ensure a thorough understanding of the study objectives and interview guides.

We have revised the Methods section to reflect that interviewers were trained in qualitative research as follows:

“Interviews were held in a private room at KCH and conducted in the local language of Chichewa by a facilitator trained in qualitative research. The interview guide was developed by the research team, reviewed by local experts, and piloted prior to interviews (Supplementary File).”

7. **Reviewer 2:** Results: In table 1. Consider using the median rather than the mean to describe the number of children. Given the limited information presented in the table, integrating these results directly into the text could improve the overall narrative and make findings more accessible.

   **Author’s response:** Thank you for this suggestion. We have updated Table 1 to report the median rather than mean for number of children, as recommended. Additionally, based on Reviewer 1’s comments, we have expanded Table 1 to include demographic information about the infants to better demonstrate the diversity of the study population.

---

We acknowledge the suggestion to integrate results directly into the text for improved accessibility, However, we believe that maintaining a structured table allows for clearer presentation of key demographic findings.
